# Effects of repetitive peripheral magnetic stimulation for the upper limb after stroke: Meta-analysis of randomized controlled trials

**DOI:** 10.1016/j.heliyon.2023.e15767

**Published:** 2023-04-22

**Authors:** Ze-Jian Chen, Yang-An Li, Nan Xia, Ming-Hui Gu, Jiang Xu, Xiao-Lin Huang

**Affiliations:** a Department of Rehabilitation Medicine, Tongji Hospital, Tongji Medical College, Huazhong University of Science and Technology, Wuhan, 430030, China; b World Health Organization Cooperative Training and Research Center in Rehabilitation, Wuhan, 430030, China

**Keywords:** Repetitive peripheral magnetic stimulation, Stroke, Upper limb, Motor recovery, Spasticity

## Abstract

**Introduction:**

Repetitive peripheral magnetic stimulation (rPMS) can stimulate profound neuromuscular tissues painlessly to evoke action potentials in motor axons and induce muscle contraction for treating neurological conditions. It has been increasingly used in stroke rehabilitation as an easy-to-administer approach for therapeutic neuromodulation.

**Objective:**

We performed this meta-analysis of randomized controlled trials to systematically evaluate the effects of rPMS for the upper limb in patients with stroke, including motor impairment, muscle spasticity, muscle strength, and activity limitation outcomes.

**Methods:**

The meta-analysis was conducted following the Preferred Reporting Items for Systematic Reviews and Meta-Analyses (PRISMA) guideline. PubMed, EMBASE, Web of Science, Cochrane Library, and Physiotherapy Evidence Database (PEDro) were searched for articles published before June 2022. Forest plots were employed to estimate the pooled results of the included studies, and the I^2^ statistical analysis was used to identify the source of heterogeneity. Publication bias was examined by Egger’s regression tests or visual inspection of the funnel plots.

**Results:**

The database searches yielded 1052 potential eligible literature; of them, five randomized controlled trials met the eligible criteria, involving a total of 188 participants. Patients in the rPMS group showed better improvement in motor impairment as measured by the FM-UE (MD: 5.39 [95% CI, 4.26 to 6.52]; *P* < 0.001; I^2^ = 0%) compared with the control group. Among the secondary outcomes, no difference was found in the improvement of muscle spasticity (SMD: 0.36 [95% CI, −0.05 to 0.77]; *P* = 0.08; I^2^ = 41%). There was a significant difference in the proximal (SMD: 0.58 [95% CI, 0.10 to 1.06]; *P* = 0.02; I^2^ = 0%) but not the distal muscle strength (SMD: 1.18 [95% CI, −1.00 to 3.36]; *P* = 0.29; I^2^ = 93%). Moreover, the activity limitation outcomes were significantly improved with rPMS intervention (SMD: 0.59 [95% CI, 0.08 to 1.10]; *P* = 0.02; I^2^ = 0%).

**Conclusion:**

This meta-analysis showed that rPMS might improve upper limb motor impairment, proximal muscle strength, and activity limitation outcomes but not muscle spasticity and distal strength in patients after stroke. Due to the limited number of studies, further randomized clinical trials are still warranted for more accurate interpretation and clinical recommendation.

## 1 Introduction

Stroke is a leading cause of adult mortality and disability, which has given rise to substantial healthcare demand and socioeconomic burden around the world [1]. Upper limb motor impairment is one of the most frequent conditions after stroke, manifesting as muscle weakness, loss of coordination, spasticity, or abnormal synergies [2,3]. It was reported that approximately 80% of the individuals with acute stroke have upper limb motor impairment. Furthermore, 50–60% of these survivors have persistent impaired function of the upper extremity 6 months after a stroke, resulting in long-term functional limitations on motor performance and activities of daily living (ADL) [4,5]. However, evidence on effective upper limb motor recovery treatments after stroke is still limited [6].

Repetitive peripheral magnetic stimulation (rPMS) is an innovative non-invasive treatment approach developed for therapeutic neuromodulation in patients with neurological conditions [7,8]. Analogous with neuromuscular electrical stimulation to some extent, rPMS can induce action potentials in the motor axons and evoke muscle contraction for the patients [9,10]. Applying the stimulation coil peripherally, rPMS can stimulate the profound neural and muscular tissues painlessly in order to increase muscle strength, alleviate spasticity, and promote motor recovery [11]. Moreover, rPMS generates proprioceptive information input during the muscle contraction-relaxation cycles that could affect the excitability of the sensorimotor system and induce neural plasticity [12,13].

Although the clinical effects of rPMS have been explored in several randomized controlled trials (RCTs), a meta-analysis of its pooled effectiveness that specifically focused on the upper limb is lacking [[14], [15], [16], [17], [18]]. The Cochrane reviews by Sakai and colleagues preliminarily found that rPMS could significantly improve ADL and functional ability but not upper limb motor function after stroke [19,20]. However, the conclusion concerning rPMS efficacy for stroke rehabilitation was limited by evidence because the number of included trials was insufficient. Therefore, the overall results regarding its effects on the upper limb after stroke remain unclear.

The aim of this meta-analysis of randomized controlled trials (RCTs) was to examine whether rPMS was effective for upper limb motor impairment, spasticity, muscle strength, and activity limitation outcomes in patients after stroke. The findings of this study may provide evidence for the application of rPMS in clinical practice and identify possible directions for further clinical trials.

## 2 Methods

The current study was performed following the Preferred Reporting Items for Systematic Reviews and Meta-Analysis guideline (PRISMA) [21]. The study protocol was prospectively registered with the PROSPERO database (www.crd.york.ac.uk/PROSPERO/; registration number: CRD42022336264).

### 2.1 Search strategy

Two review authors (ZJC and YAL) conducted independent computerized literature searches of the following databases for potential articles: PubMed, EMBASE, Web of Science, Cochrane Library, and Physiotherapy Evidence Database (PEDro) from inception until June 30, 2022. In consultation with an academic librarian, the search strategy for the PubMed database focused on the following key terms: “repetitive peripheral magnetic stimulation”, “upper limb”, and “stroke”; and were modified to be adapted for the other electronic databases (Supplemental Table S1). Moreover, a manual search in the reference lists of the included articles was carried out to identify additional literature.

### 2.2 Study selection

The retrieved articles were initially imported into the EndNote X7 software, and the duplicates of publications were removed subsequently. Two independent reviewers (NX and MHG) were responsible for screening the titles as well as abstracts of all retrieved articles. After accounting for and eliminating the publications that were not relevant to rPMS intervention for patients with stroke, the remaining full-text articles were assessed against the eligibility criteria to identify potential studies. When necessary, disagreements regarding study selection would be determined by consultations with the third study reviewer (XLH).

### 2.3 Eligibility criteria

#### 2.3.1 Types of studies

We included RCTs that investigated the rPMS effects for the upper limb in individuals after stroke. Only studies published in English would be included. We excluded the articles that were nonrandomized trials, protocols, letters, and animal studies. Furthermore, studies without sufficient data for the primary or secondary outcomes, full-text, or sufficient quality (as measured by PEDro scoring below 4) were excluded.

#### 2.3.2 Types of participants

Adult participants (≥18 years) with upper limb motor impairment due to stroke diagnosed using neuroimaging examination (CT or MRI), regardless of their clinical characteristics, including sex, age, stroke type, severity, and disease duration. Studies with mixed participants consisted of stroke individuals and other acquired brain injuries, such as traumatic brain injury, were considered if most of the participants had a diagnosis of stroke (Supplemental Table S2).

#### 2.3.3 Types of interventions

Non-invasive rPMS that targeted the upper limb muscles, nerves, or spinal roots, instead of the central nervous system (brain or spinal cord) [19].

#### 2.3.4 Types of comparisons

All studies that compared rPMS with sham intervention or usual care, with or without conventional rehabilitation therapy for the upper extremity, were included in the current meta-analysis.

#### 2.3.5 Types of outcome measures

The primary outcome of the meta-analysis was motor impairment measured by the Fugl-Meyer Assessment of the Upper Extremity (FM-UE) [22]. Secondary outcomes of interest were based on the evaluation of muscle spasticity, muscle strength, and activity limitation. Muscle spasticity was measured using the Modified Ashworth Scale (MAS) or the Modified Tardieu Scale. Muscle strength was measured by the Manual muscle test (MMT), surface electromyography (sEMG), or the Medical Research Council (MRC) scale. Activity limitation was assessed by either the Barthel Index (BI), Functional Independence Measure (FIM), Wolf Motor Function Test (WMFT), Action Research Arm Test (ARAT), Box and Blocks Test (BBT), or other available scales [23]. If multiple outcomes were used for the same outcome, only one was allowed to be included in the pooled results.

### 2.4 Data extraction

Two reviewers (ZJC and YAL) extracted information independently from the included studies with a standardized data extraction form. Moreover, a third auditor (JX) crosschecked the retrieved data to ensure accuracy. Information extraction was composed of the author, year of publication, study design, patients'’ characteristics (age, gender, stroke type and severity, time since onset of stroke, sample size), methodological quality (PEDro scale), intervention details (frequency, intensity, stimulation session, duration, coil design, and treatment target) and outcome measures. If information and data were not accessible, we contacted the corresponding authors of relevant publications via email.

### 2.5 Assessment of risk of bias and methodological quality

Two reviewers (ZJC and YAL) were involved in the risk of bias (quality) assessment independently. The risk of bias in each study was evaluated using the Cochrane risk of bias assessment tool and was rated as high, low, or unclear risk. For this study, the PEDro scoring system was used to assess the methodological quality and risk of bias for the included studies [24]. Studies with a PEDro score of four points or more (sufficient quality) would be included, otherwise (insufficient quality) would be excluded from the pooled estimates [25]. Any uncertainty or disagreement was resolved by discussion with the third reviewer (JX) for a final decision.

### 2.6 Statistical analysis

The software RevMan 5.3 was used for statistical analysis. Means with standard deviations of the change scores for the outcomes were extracted in the rPMS and control groups to estimate 95% confidence intervals (CIs). For the primary outcome, mean difference (MD) with 95% CI was calculated to determine the treatment effect. For the secondary outcomes, standardized mean differences (SMD) with 95% CIs were used since different outcome scales were reported.

Forest plots were employed to graphically represent the pooled results of the included studies. We analyzed the source of heterogeneity using the I^2^ statistical analysis [26]. If I^2^ ≥ 50%, substantial statistical heterogeneity was reported and the continuous variable random-effect model was utilized for data analysis. Otherwise, low heterogeneity was considered and a fixed effect model was used afterward. To address high heterogeneity, the reliability of meta-analysis results was evaluated with the prespecified sensitivity analyses by omitting individual studies and inspecting the result robustness. Publication bias was assessed by Egger’s regression tests or visual inspection of the funnel plots [27]. Data were analyzed according to the intention-to-treat principle. All *P* values were 2-tailed with statistical significance specified at 0.05.

## 3 Results

**Fig. 1:**
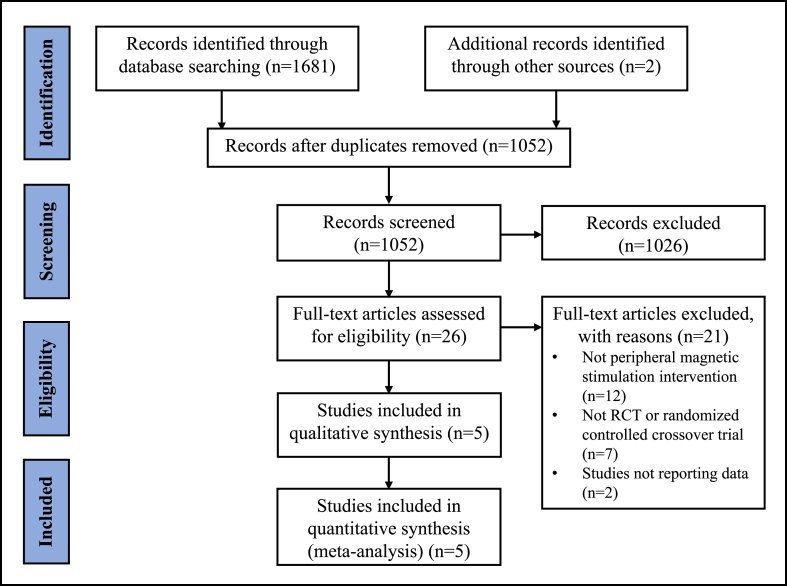
PRISMA flow chart.

A PRISMA flow chart representing the literature retrieval process of the meta-analysis was shown in Fig. 1. The database searches yielded a total of 1683 references for screening. After removing all duplicates, 1052 references were remaining for detailed screening of titles and abstracts. After review, five randomized controlled trials of rPMS intervention met the study entry criteria, involving a total of 188 participants.

### 3.1 Description of the included studies

Characteristics of the included trials are presented in Table 1. Of the included studies, the mean age ranged from 41.6 to 72.3, and 63.3% of the participants were male. rPMS protocols of the studies were heterogeneous, with 20–50 Hz frequency, 600–5000 pulses, 15–30 min, and 8–20 sessions of magnetic intervention. Two studies used muscle contraction threshold [14,16] and there used maximal stimulator output to determine the treatment intensity [15,17,18]. Moreover, magnetic coils were placed mostly on the extensors and flexors of the upper extremity [[14], [15], [16], [17]]; one was applied to the axilla for stimulation of the brachial plexus [18].

**Table 1:** Characteristics of the included studies.

Study Name	Sample size	Age (E/C) Mean ± SD/Median [IQR] (y)	Sex (male/female)	Stroke duration (E/C) Mean ± SD/Median [IQR]	Stroke type (ischemic/hemorrhage)	Frequency, Intensity, No. of pulses	On/Off (s)	Duration per session; Treatment duration	Coil type	Site	Control Intervention	Outcome Measures	Additional Intervention
Jiang 2022	24/20	54.62 ± 10.89 56.09 ± 16.59	27/17	13.81 ± 2.51 days 14.45 ± 3.33 days	32/12	20 Hz, 15–30% MSO, 2400	0.5/2	20 min; daily, 14 days	Round	Triceps brachii and extensor digitorum muscles	No treatment	FM-UE BI RMS	Conventional physiotherapy
Ke 2022	13/13	58 (46.5–63.0) 56 (46.5–61.5)	14/12	17 (8.0–42.5) days 16 (4.5–22.0) days	0/26	20 Hz, 40–60% MSO, 1800	1/19	30 min; daily, 10 days	Figure-of-eight	Axilla	Sham	FM-UE MRC	Conventional treatments
Krewer 2014	31/32	55 ± 13 54 ± 13	38/25	26 ± 71 weeks 37 ± 82 weeks	60 stroke/3 traumatic brain injury	25 Hz, 10% above MCT, 5000	1/2	20 min; 2/day, 5/week, 2 weeks	Figure-of-eight	Extensors and flexors of the upper arm	Sham	FM-UE MTS	Occupational therapy
Nahas 2022	25/11	47.88 ± 14.8 41.60 ± 14.9	27/9	42.74 ± 52.74 months 64.09 ± 67.07 months	23 stroke/13 other diseases	50 Hz, Above MCT, 600	2/8	1600 s; Daily, 8 days	Figure-of-eight	Biceps brachii, flexors of the wrist and fingers	Sham	MAS eBTD	–
Obayashi 2020	10/9	64.3 ± 13.1 72.3 ± 10.7	13/6	9.2 ± 4.4 days 5.8 ± 2.2 days	16/3	30 Hz, 70% MSO, Not mentioned	2/2	15–20 min; Daily, 5/week until transfer	Round	Deltoid, triceps brachii, extensor carpi radialis and extensor digitorum communis; biceps brachii and flexor digitorum superficialis (if applicable)	Standard care	FM-UE WMFT BBT	Standard care

BBT, box and block test; BI, Barthel Index; FM-UE, the upper-extremity motor section of the Fugl-Meyer Motor Assessment Scale; eBTD: estimated Botulinum toxin dose; MAS, Modified Ashworth scale; MCT, muscle contraction threshold; MRC, Medical Research Council scale; MSO, maximal stimulator output; MTS, Modified Tardieu Scale; WMFT, Wolf motor function test.

### 3.2 Risk of bias and methodological quality of the included studies

The results of the risk of bias summary and graph were shown in Fig. 3 (A, B). The trials were of high quality on randomization, allocation concealment, and outcome data. Patients were not blinded in two studies [15,17], and one study did not mention the blinding of outcome assessor [17]. Overall, the included studies demonstrated moderate-to-high quality as indicated by the PEDro scoring system (Table 2). Quality deficits were mainly derived from the blinding process and insufficient follow-up period.

**Table 2:** PEDro scores of the included studies.

Study	Random allocation	Concealed allocation	Baseline comparability	Blind subjects	Blind therapists	Blind assessors	Adequate follow-up	Intention-to-treat analysis	Between-group comparisons	Point estimates and variability	Total score (0–10)
Jiang 2022	1	1	1	0	1	1	0	1	1	1	8
Ke 2022	1	1	1	1	1	1	0	0	1	1	8
Krewer 2014	1	1	1	1	1	1	1	1	1	1	10
Nahas 2022	1	1	1	1	1	1	0	1	1	1	9
Obayashi 2020	1	0	1	0	0	0	0	1	1	1	5

### 3.3 Primary outcome

**Fig. 2:**
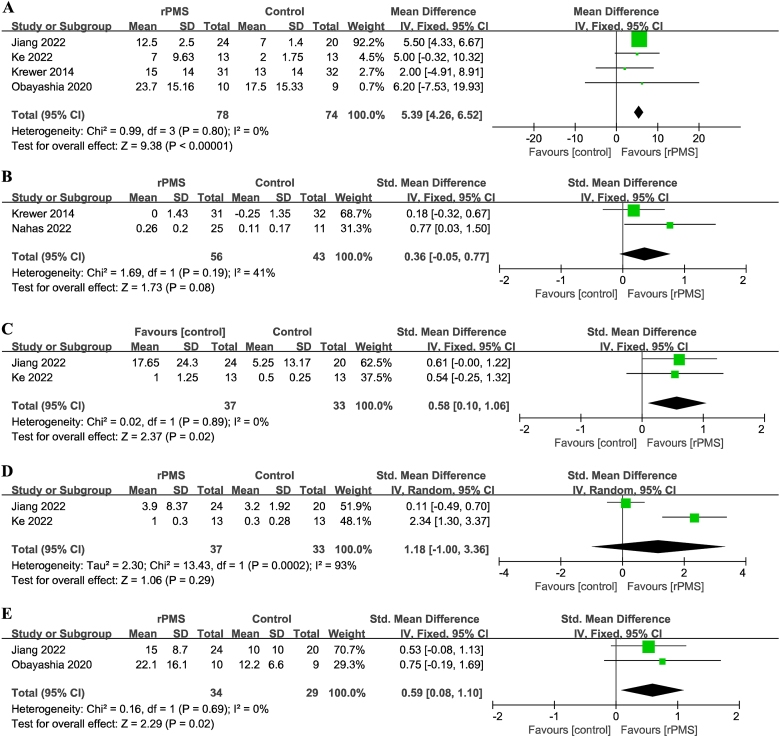
Forest plots of the pooled outcomes. (A) Motor function: *P* < 0.001; I^2^ = 0%. (B) Spasticity: *P* = 0.08; I^2^ = 41%. (C) Proximal muscle strength: *P* = 0.02; I^2^ = 0%. (D) Distal muscle strength: *P* = 0.29; I^2^ = 93%. (E) Activity limitation outcomes: *P* = 0.02; I^2^ = 0%. rPMS, repetitive peripheral magnetic stimulation.

For the primary outcome, motor impairment as measured by the FM-UE, data were available from 4 trials involving 162 participants [14,15,17,18]. Patients in the rPMS group showed better improvement (mean difference [MD], 5.39 [95% CI, 4.26 to 6.52]; *P* < 0.001; I^2^ = 0%; fixed-effects model; [Fig. 2A]) compared with the control group significantly.

**Fig. 3:**
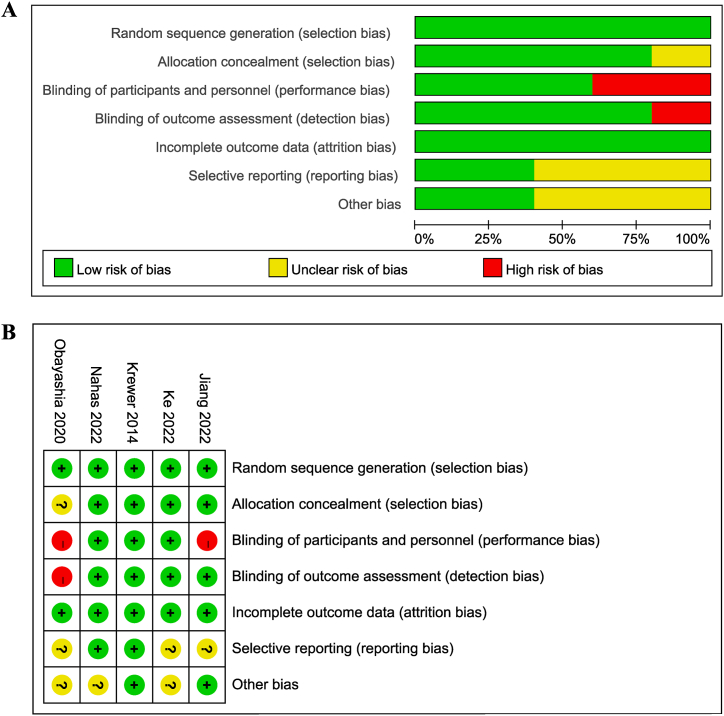
Risk of bias assessment using the Cochrane tool. (A) Risk of bias summary. (B) Risk of bias graph.

### 3.4 Secondary outcomes

Among the secondary outcomes assessed, we found no statistical difference in the recovery of muscle spasticity (SMD, 0.36 [95% CI, −0.05 to 0.77]; *P* = 0.08; I^2^ = 41%; 2 trials; 99 participants; [Fig. 2B]) [14,16]. There was a significant difference in the proximal muscle strength (SMD, 0.58 [95% CI, 0.10 to 1.06]; *P* = 0.02; I^2^ = 0%; 2 trials; 70 participants; [Fig. 2C]) [15,18], but not in the distal muscle strength (SMD, 1.18 [95% CI, −1.00 to 3.36]; *P* = 0.29; I^2^ = 93%; 2 trials; 70 participants; [Fig. 2D]) [15,18]. Moreover, patients treated with rPMS had significant improvement in activity limitations (SMD, 0.59 [95% CI, 0.08 to 1.10]; *P* = 0.02; I^2^ = 0%; 2 trials; 63 participants; [Fig. 2E]) [15,17].

The degree of heterogeneity of the included RCTs was generally low. However, sensitivity analysis indicated that the result on distal muscle strength should be interpreted with caution. Due to the limited number of RCTs included, Egger’s tests were unable to be performed. There was low publication bias for the assessed outcomes by visual inspection of the funnel plots (Supplemental Fig. S1).

## 4 Discussion

The current study preliminarily supported the clinical application of rPMS for the upper limb in post-stroke survivors. The pooled results demonstrated that rPMS, compared with sham or no treatment, significantly improved upper limb motor impairment. Limited evidence suggested rPMS treatment was associated with improved proximal arm muscle strength and functional activity, and similar outcomes for distal arm muscle strength and spasticity. To our best knowledge, this is the first meta-analysis of randomized controlled trials focused on evaluating the rPMS effects on post-stroke rehabilitation for the upper extremity.

As a non-invasive and painless neuromodulation approach, rPMS enables straight stimulation of the profound neuromuscular structures that cannot be reached by general electrical stimulation for post-stroke survivors. Moreover, rPMS could generate proprioceptive feedback to the central nervous system during peripheral muscle contraction. According to previous literature, optimization of neuromuscular activity at the treatment locations, synaptic strengthening of the spinal cord, neural activation of the cerebral cortex via afferent input, or regulation of the corticospinal motor excitability, are the possible mechanisms of action for rPMS treatment in individuals after stroke [7,15].

Four studies in the meta-analysis examined the rPMS effects on motor function, three of which included participants with early stroke. In line with our results, Obayashi and colleagues found that rPMS to the triceps brachii and extensor digitorum muscles, compared with standard care, improved upper extremity function and functional capacity in patients most with acute ischemic stroke [17]. The protocol of administering rPMS to the arm extensors combined with conventional physical therapy could have also improved upper limb function in the patients. Following rPMS intervention, Struppler and colleagues found neural activation of the parietal-premotor network that was associated with motor recovery [28]. Additionally, Ke et al. applied high-frequency rPMS to the brachial plexus that enhanced motor function (measured by FM-UE scores) and the proximal muscle strength (measured by MRC scores) of the upper extremity in participants with intracerebral hemorrhage [18]. However, Krewer et al. reported contradictory results that rPMS intervention on both the extensors and flexors had no significant improvement on motor function in 60 participants with chronic stroke and three with traumatic brain injury [14]. The pooled results indicated that disease duration from stroke onset could have influenced the treatment effect on upper limb motor function. During the early stage after stroke, spontaneous recovery was composed of synaptogenesis, sprouting of new dendrites and axons, as well as migration of neural stem cells [29,30]. The results suggested that rPMS application may have coupling effects for cortical reorganization and neural regeneration in this plastic window.

The pooled results suggested that rPMS was beneficial to the recovery of the proximal muscle strength but not to the distal. Among the two studies evaluating outcomes concerning muscle strength, the included participants were of severe upper limb paralysis. Performing magnetic stimulation peripherally, the intervention can induce direct activation of skeletal muscles and sensorimotor nerves, thus producing information input and promoting cerebral excitability [15]. However, the recovery of hand strength was slow for the patients with such an extent of motor impairment during the early stroke. It should be noted that heterogeneity was high in the meta-analysis of the distal strength. Other clinical factors such as the difference in stimulation locations and age/gender of the participants can also lead to heterogenous outcomes. Therefore, further studies should clarify the relationship between coil locations, such as the superficial muscles (extensors or flexors, agonistic or antagonistic muscles), nerves, or spinal roots, and motor recovery in various demographic characteristics of patients. Importantly, researchers need to carefully consider how the recruitment of focal neuromuscular tissues can be affected by coil types to prevent unwanted stimulation around the intervention sites.

The included RCTs did not provide sufficient information for the remaining outcomes, especially for the activity limitation outcomes. As a result, future large-scale studies are needed to verify the effectiveness of rPMS on these outcomes. For muscle spasticity, Krewer et al. found that rPMS showed effectiveness only for the wrist flexors, but not for the other muscles. Although the current study lacks sufficient evidence for muscle spasticity, the significant difference in their study was mainly derived from a deterioration in the control group with sham stimulation. It was speculated that rPMS may have prevented an increase in muscular spasticity because the patients had lower MAS values at baseline while the treatment was more pronounced with higher grades of spasticity. The technique of rPMS stimulation may be of importance as well. For example, Nahas et al. demonstrated that peripheral intermittent theta burst stimulation could improve spasticity on hypertonic muscles and a consequent significant reduction in the estimated Botulinum toxin dose [16]. Spasticity was characterized by hypertonic stretch reflexes resulting from abnormal control of the descending brainstem pathways, which was closely associated with motor recovery [31]. Since the cortical spinal tract and reticulospinal tract were regulated by the cerebral cortex, the lesion location of the stroke, such as the internal capsule and basal ganglia, can influence the effect of rPMS on motor function and spasticity, which were unfortunately not introduced in the two studies. Moreover, the type of stroke matters for the patients because it was related to different symptoms and recovery mechanisms [32,33].

Several limitations in the present meta-analysis should be noted to carefully interpret the results. First, even if the included RCTs demonstrated low risk of bias and moderate-to-high PEDro scores, the small number of available studies may limit the findings. Second, the included trials were limited by insufficient follow-up durations, thus the long-term rPMS effects for the upper limb in stroke individuals should be evaluated in the future. Finally, the variability presented in the clinical characteristics of the participants, rPMS protocols (frequency, intensity, stimulation session, duration, coil design, and treatment target), and outcome measures among the included single-center trials may lead to heterogeneities and limit the certainty of the evidence. Additional multi-center, high-quality studies with sufficient sample sizes are required to determine further conclusions.

## 5 Conclusions

In conclusion, this meta-analysis demonstrated that repetitive peripheral magnetic stimulation was beneficial in improving upper limb motor impairment in people after a stroke. The study revealed insufficient evidence that rPMS might be effective for proximal muscle strength and the activity limitation outcomes, but not to muscle spasticity and distal strength. Considering there were not enough trials contributing to the meta-analyses, larger, high-quality randomized controlled trials with follow-up assessments are still required to determine further conclusions.

## Author contribution statement

All authors listed have significantly contributed to the development and the writing of this article.

## Data availability statement

Data included in article/supplementary material/referenced in article.

## Declaration of interest’s statement

The authors declare that they have no known competing financial interests or personal relationships that could have appeared to influence the work reported in this paper.
