# Supplementary material for: Effects of repetitive peripheral magnetic stimulation for the upper limb after stroke: Meta-analysis of randomized controlled trials
Source: Heliyon. 2023 Apr 22;9(5):e15767. doi: 10.1016/j.heliyon.2023.e15767 (PMC10172780; doi:10.1016/j.heliyon.2023.e15767)
Supplement: Multimedia component 2 [file mmc2.docx]

**SUPPLEMENTAL MATERIAL**

**Table and Figure Legends**

- Table S1: Search strategy for PubMed.
- Table S2. Eligible criteria for the participants of the included studies.
- Figure S1-A: Funnel plot for publication bias assessment of motor function.
- Figure S1-B: Funnel plot for publication bias assessment of spasticity.
- Figure S1-C: Funnel plot for publication bias assessment of proximal muscle strength.
- Figure S1-D: Funnel plot for publication bias assessment of distal muscle strength.
- Figure S1-E: Funnel plot for publication bias assessment of activity limitation outcomes.

**Table S1. Search strategy for PubMed.**

|  | **Search items** |
| --- | --- |
| AND | ("Upper extremity"[All Fields] OR "Arm"[All Fields] OR "Elbow"[All Fields] OR "Forearm"[All Fields] OR "Hand"[All Fields] OR "Wrist"[All Fields] OR "Shoulder"[All Fields] OR "Finger"[All Fields] OR "peripher*"[All Fields] OR "nerv*"[All Fields] OR "musc*"[All Fields] OR "spasti*"[All Fields] OR "hemipleg*"[All Fields] OR "hemipar*"[All Fields] OR "paresis*"[All Fields]) |
| AND | ("Stroke"[Mesh] OR ("stroke"[MeSH Terms] OR "stroke"[All Fields] OR ("cerebrovascular"[All Fields] AND "accident"[All Fields]) OR "cerebrovascular accident"[All Fields]) OR ("stroke"[MeSH Terms] OR "stroke"[All Fields]) OR (chronic [All Fields] AND ("stroke"[MeSH Terms] OR "stroke"[All Fields]))) |
| AND | (“Magnetic Field Therapy” [Mesh] OR “repetitive peripheral magnetic stimulation” [All Fields] OR “peripheral magnetic stimulation” [All Fields] OR “PMS” [All Fields] OR “rPMS” [All Fields]) |
| AND | English[lang] |

| **Study** | **Inclusion criteria** | **Exclusion criteria** |
| --- | --- | --- |
| **Jiang 2022** | (1) First-ever unilateral ischemic or hemorrhagic stroke in the basal ganglia within 1-4 weeks; (2) medically stable; (3) age 30–80 years; (4) a Brunnstrom Stage of 1-2 for the upper limb and hand; and (5) provide written informed consent. | (1) Severe spasticity of an upper extremity, with a score of more than three on the Modified Ashworth scale (MAS); (2) severe aphasia or cognitive impairment that could prevent informed consent or interfere with the study’s behavioral measurements; (3) infection near the stimulation site; (4) deep-vein thrombosis near the stimulation site; (5) unstable fractures of the paretic upper extremity; (6) any contraindications to rPMS (e.g. metal implants in the affected limb or use of a pacemaker); or (7) use of muscle relaxants such as baclofen or tizanidine or injections with Botulinum Toxin A for post-stroke spasticity. |
| **Ke 2022** | Patients who were 18-70 years old, within 8 weeks of first-ever intracerebral hemorrhage onset, conscious and had stable vital signs. Written signed informed consent was obtained from all participants. | Ineligible if the intracerebral hemorrhage was attributable to head injury; had other central nervous system diseases (such as ischemic stroke, subarachnoid hemorrhage, and subdural hemorrhage); were combined unstable arrhythmia, fever, infection and epilepsy; were severe aphasia or cognitive impairment leading to poor cooperation; with contraindications for magnetic stimulation or severe limb orthopedic disease. |
| **Krewer 2014** | (1) Hemiparesis caused by a stroke or a traumatic brain injury; (2) spasticity of an upper extremity, with a score of 1-3 on the Tardieu Scale; and (3) ages 18-75 years. | (1) Metal implant in the head or within the stimulation area; (2) medical implanted devices (cardiac pacemaker, cochlea implant, or medication pumps); (3) pregnancy; (4) comorbidity with other neurodegenerative disorders or other neurologic, orthopedic disorders; (5) increased intracranial pressure; and (6) unstable fractures of the paretic upper extremity. |
| **Nahas 2022** | Age more than 18 yrs., disease duration > 6 months with persistent spasticity in the affected muscle (≥1+ by MAS) and no change in anti-spasticity medications for at least one month prior to recruitment. | Recent Botulinum toxin injection for limb spasticity (<4 months), a metal plate along the spastic limbs, patients with pacemakers and pregnant females. |
| **Obayashia 2020** | (1) Medically stable; (2) hospitalized adults within 2 weeks of stroke; (3) age 20–89 years; (4) intact skin on the hemiparesis arm; (5) adequate cognition to participate; (6) first-ever, severe UE paresis (stroke impairment assessment set (SIAS): knee-mouth test range 1–3; finger function test 1a-3). | (1) Past history of stroke, brain injury or brain tumor; (2) metal implant in head or within stimulation area; (3) dysphasia; (4) history of cardiac arrhythmia with hemodynamic instability; (5) comorbidity with neurodegenerative diseases and mental disorders; (6) uncontrolled seizure disorder; (7) disturbed consciousness; (8) implanted stimulator (such as cardiac pacemaker); (9) pregnancy. |

**Table S2: Eligible criteria for the participants of the included studies.**

**
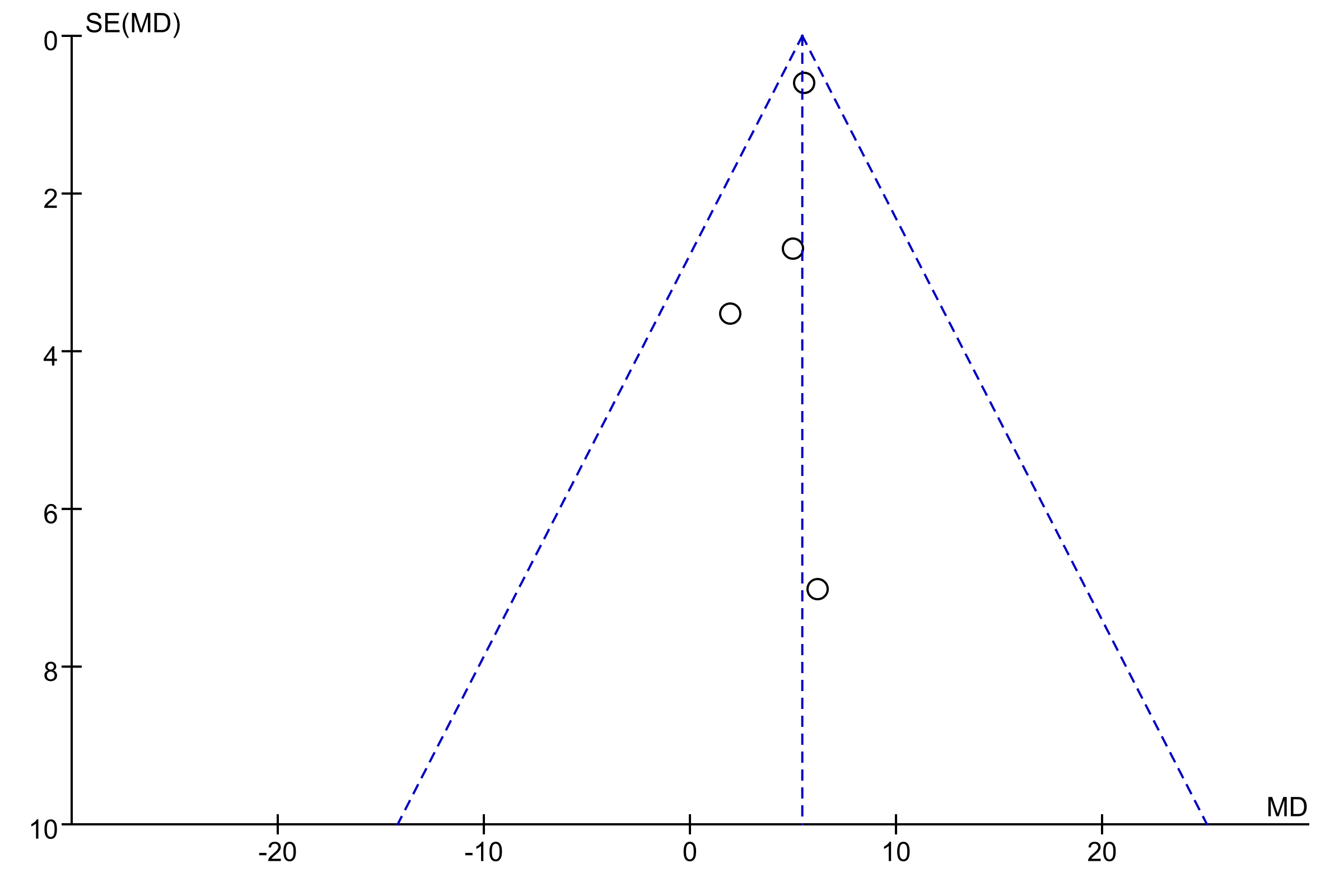
**

**Figure** **S1-A: Funnel plot for publication bias assessment of motor function.**

**
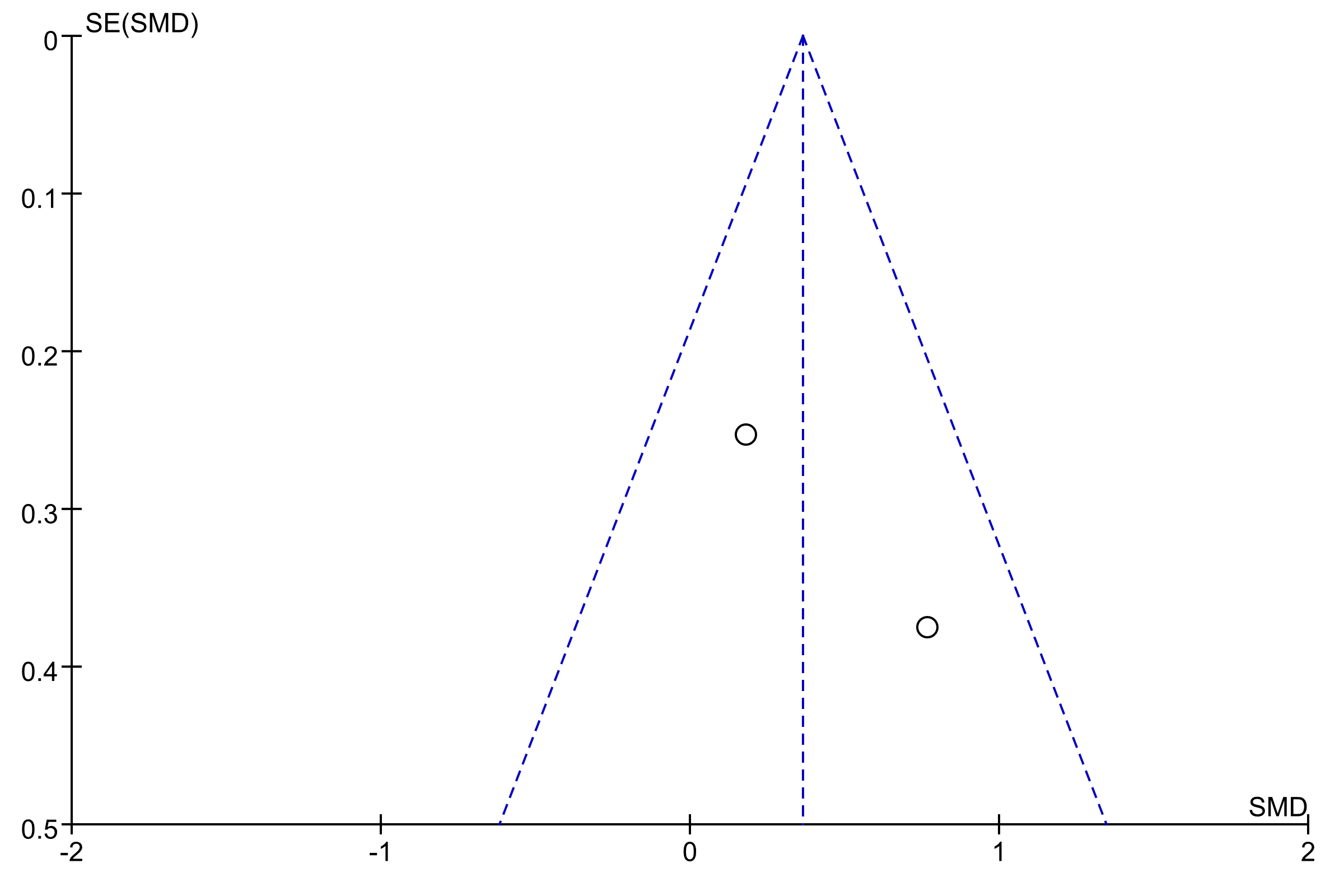
**

**Figure S1-B: Funnel plot for publication bias assessment of spasticity.**

**
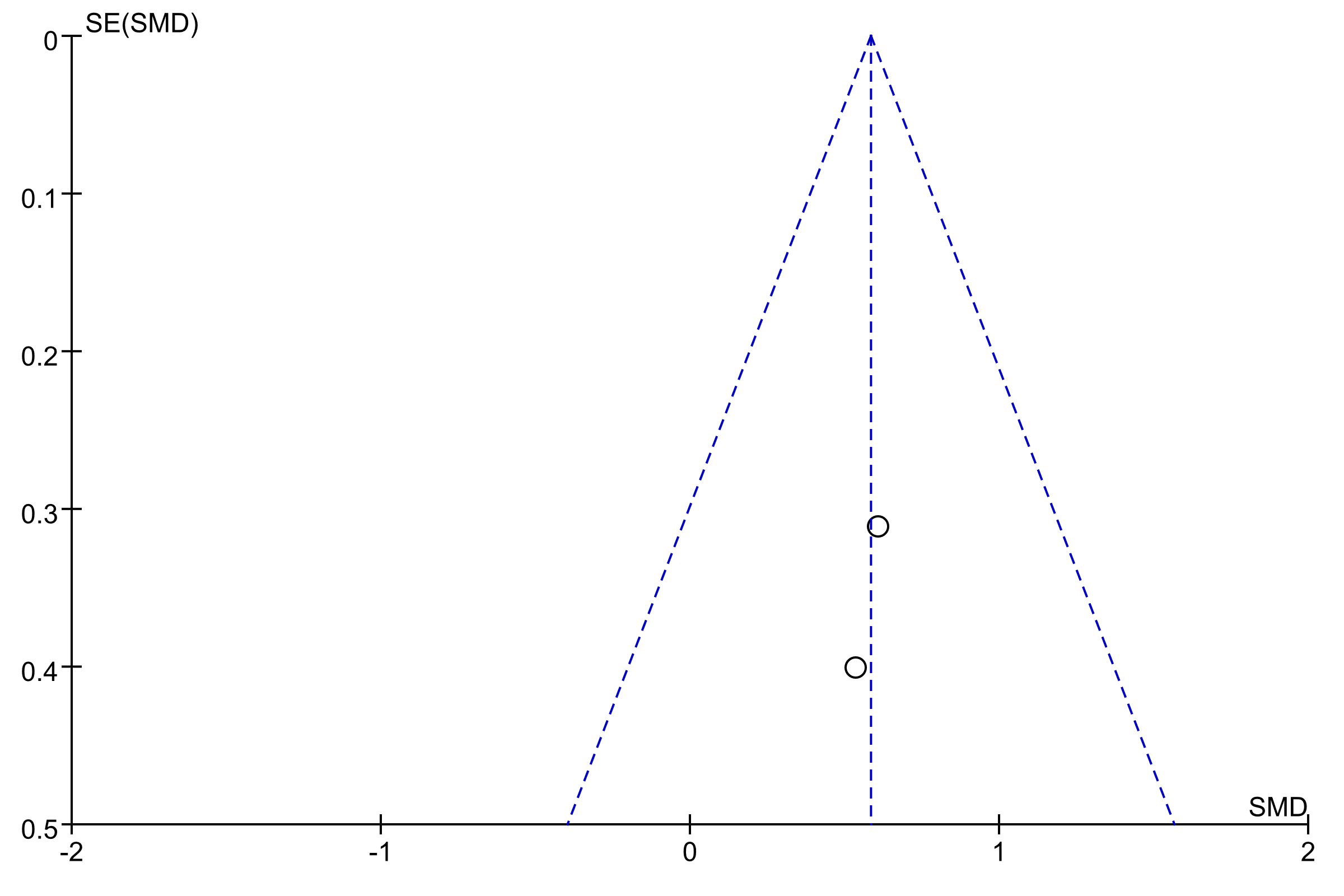
**

**Figure S1-C: Funnel plot for publication bias assessment of proximal muscle strength.**

**
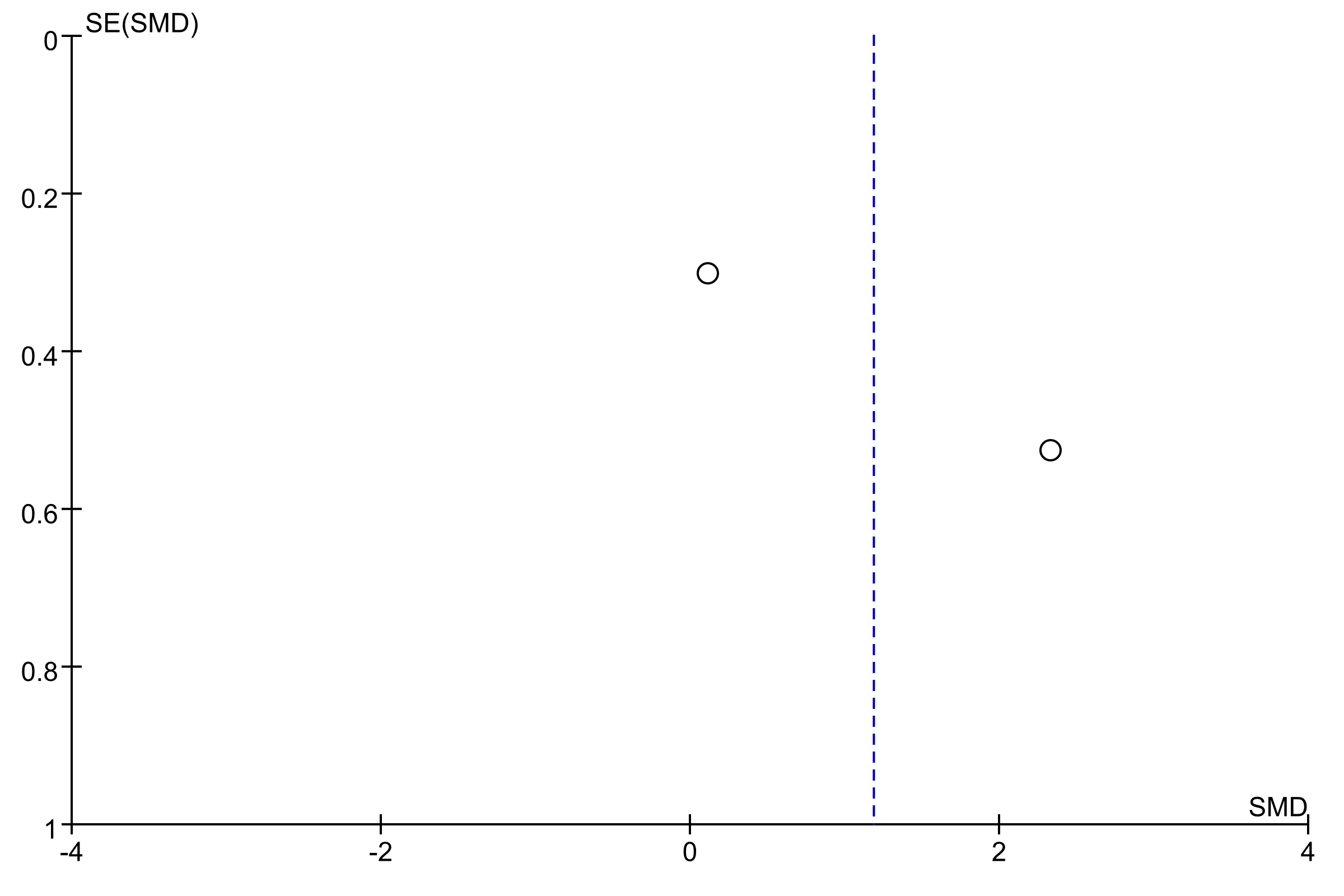
**

**Figure S1-D: Funnel plot for publication bias assessment of distal muscle strength.**

**
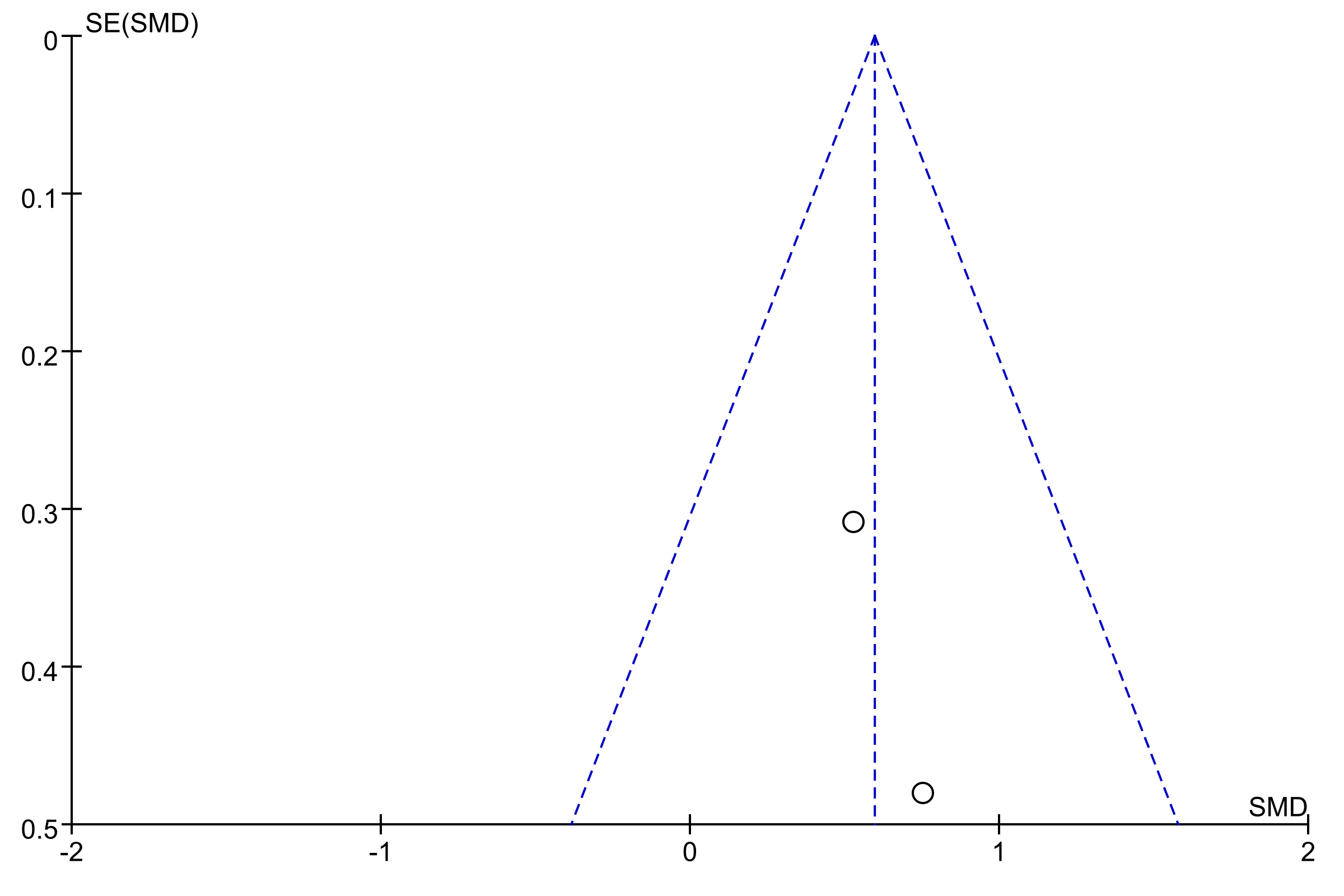
**

**Figure S1-E: Funnel plot for publication bias assessment of activity limitation outcomes.**
